# Infant, pediatric and adult well visit trends before and during the COVID-19 pandemic: a retrospective cohort study

**DOI:** 10.1186/s12913-022-07719-7

**Published:** 2022-03-11

**Authors:** Joanne Salas, Leslie Hinyard, Ann Cappellari, Katie Sniffen, Christine Jacobs, Natalie Karius, Richard A. Grucza, Jeffrey F. Scherrer

**Affiliations:** 1 Department of Family and Community Medicine, Saint Louis University School of Medicine, 1008 S. Spring, St. Louis, MO 63110 U.S.A.; 2 Advanced HEAlth Data (AHEAD) Research Institute, Saint Louis University School of Medicine, 3545 Lafayette Ave, 4th Floor, St. Louis, MO 63104 U.S.A.; 3 Department of Health and Clinical Outcomes Research, Saint Louis University School of Medicine, 3545 Lafayette Ave, 4th Floor, St. Louis, MO 63104 U.S.A.; 4 Department of Integrated Health Technology, SSM Health Care, 7980 Clayton Rd, St. Louis, MO 63117 U.S.A.

**Keywords:** COVID-19, Pandemic, Well visit, Prevention, Primary care, Pediatrics, Family medicine, Cohort

## Abstract

**Background:**

Adult well visits declined during COVID-19, but literature is inconsistent in regard to whether childhood well visits declined. We determined if the COVID-19 pandemic was associated with a change in well visits among infants, children, adolescents and adults before, compared to during the COVID-19 pandemic, including through the emergence of the Delta variant.

**Methods:**

De-identified electronic health care data came from a multi-state Midwest health care system. Eligible patients (*n* = 798,571) had ≥ 1 well visit between 7/1/2018 and 6/30/2021. Trends in well visits per month for children (< 1, 1–4, 5–11, 12–17 years) and adults (18–39, 40–64, ≥ 65 years) over 3-years were assessed using Joinpoint regression models and monthly percent change (MPC).

**Results:**

Well visits remained stable for infants (< 1 year of age) (MPC = -0.1; 95% CI = -0.3, 0.1). For children 1–4 years and all adults, visits were stable prior to 2020, decreased from 1/2020 to 4/2020 (MPC range -20 to -40), increased from 4/2020–7/2020 (MPC range 30 to 72), and remained stable after 7/2020. Children 5–17 had seasonal variation in visits where low points occurred in Jan/Feb 2019 and high points in Aug 2019 (start of school year); however, the low point in 2020 occurred in April 2020 and the seasonal variation normalized after this.

**Conclusions:**

In a large Mid-western health care system, infant well visits did not decline at the onset (3/1/2020) of the COVID-19 pandemic. Although well visits for all other ages decreased to a low point in 4/2020, a rapid return to pre-pandemic utilization rates occurred by 7/2020. The brief decrease in preventive care may have had little impact on health.

**Supplementary Information:**

The online version contains supplementary material available at 10.1186/s12913-022-07719-7.

## Introduction

In the United States, primary care visits dropped dramatically across different health care systems after the March 2020 stay at home orders were released in response to the COVID-19 pandemic [1–5]. Similar decreases in health care utilization were observed in other countries, including Germany, Canada, and Switzerland [6–8].

Many studies have focused on the effect of the pandemic on well-child visits; however, the literature is inconsistent regarding whether the COVID-19 pandemic interrupted well visits and immunization for patients under 2 years of age. Analysis of medical record data from over 70 pediatric clinics in the Chicago area and from German pediatric clinics revealed large decreases (e.g. 50%) in visits at the start of the stay-at-home orders [3, 6]. Immunization only/well-child encounters dropped off in March 2020 [3, 9], but evidence indicates a rapid return of up to 90% of prior utilization rates compared to the same period in 2019 [3]. A study using data from four academic medical centers in the United States revealed younger versus older children were less likely to have a large decrease in well visits [2]. This is consistent with a Canadian sample in which there was little change in well-child visits among infants less than 18 months old during the early phase of the COVID-19 pandemic as compared to 2019 utilization rates [7]. In contrast, a Santoli and colleagues study [5], using the United States Vaccines for Children Program data, revealed decreased use of immunization and well visits in March 2020 and increased utilization the following month among children under 2 years of age; however, visits for older children did not demonstrate a similar rapid increase during this same time frame.

There is more consistent evidence for an association between the COVID-19 pandemic and decreased well visits among adults [1, 4, 8]. During the shutdown, compared to a similar time period in 2019, overall encounters in Swiss general practices dropped by 17.2% and visits for HbA1c and blood pressure checks dropped by 30% to 35% [8]. Comparison of United States medical claims data between March–April 2020 versus March–April 2019, indicated that adults had ≥ 90% decrease in use of colonoscopy, mammogram and HbA1c tests but only a 7.4% and 3.5% decrease in use of chemotherapy and labor and delivery, respectively [4].

While numerous studies have examined trends in health care utilization in the immediate post-lock-down period (3/2020–4/2020), we are unaware of literature on trends in well visits a year or longer after lock-down. In addition, existing literature has not differentiated well visit utilization within HEDIS defined age groups (< 1, 1–4, 5–11, 12–17, 18–39, 40–64 and ≥ 65 years of age). HEDIS guidelines suggests different frequency of visits for each age group with younger ages requiring more frequent well visits. To elucidate long-term changes in well visits before and during the COVID-19 pandemic, we used medical record data from a large Mid-western health care system to assess well visit utilization from 7/1/2018 through 6/30/2021. We computed utilization within each age group and conducted sub-group analyses by gender and race. Our objective was to evaluate patterns of change in well visit use prior to COVID-19, during the shut-down and throughout the emergence and dominance of the Delta variant in the following 15 months.

## Methods

This research was performed in accordance with the Declaration of Helsinki. The Saint Louis University Ethics Committee, called the Saint Louis University Institutional Review Board, deemed this work to be exempt from requiring ethics approval and deemed this work as non-human subjects research because data was de-identified, retrospective and patients did not actively participate.

De-identified medical record data were obtained from the Saint Louis University-SSM HealthCare System’s Virtual Data Warehouse (VDW), a database developed from electronic health records following removal of patient and provider identifiers. The VDW captures clinical encounter data starting in 1/1/2008 from academic and non-academic ambulatory and inpatient settings in a Mid-western, multi-state health care system. The health care system covers rural and urban locations from the southern half of Wisconsin, Southern Illinois, the St. Louis, Missouri metropolitan area, mid-Missouri, and the Oklahoma City, Oklahoma metropolitan areas.

The VDW includes patients from birth to > 90 years of age who have private or public health insurance as well as uninsured who utilize health care services within the SSM Health system. The VDW is updated monthly and includes over 10.3 million unique patients who had at least one encounter in the health care system. As a member site of the Health Care Systems Research Network (www.hcsrn.org), VDW variables are defined according to HCSRN specifications. VDW variables are created from ICD-9 and ICD-10 codes, current procedural terminology (CPT) codes, pharmacy orders, laboratory orders and results, vital signs, provider type, clinic type, referrals, and demographics.

### Eligibility

Patients were eligible if they had a new patient well visit or one or more established patient well visits anytime between 7/1/2018 to 6/30/2021 (*n* = 798,571 patients). Our objective was to evaluate changes in well-visits at the system level. We did not intend to follow a specific group of patients over time. Our approach allowed us to capture use by new patients, often entering the health care system for pregnancy and subsequent pediatric well-visits.

Age was defined at each visit for each patient and the distribution calculated among all well visits. We used CPT and ICD-9/10 V and Z codes for age-group specific new patient or established patient well visits (see supplementary e-Table 1). Other descriptive sample variables, calculated at the patient level, included gender, race, and average number of visits per patient.

### Outcome

The outcome variable of interest was the total number of well visits in each month from 7/1/2018 through 6/1/2021.

### Analytic approach

To analyze temporal trends and identify changes in trends in the number of well visits per month before COVID-19 and during COVID-19, a joinpoint regression analysis on the log-transformed monthly counts was conducted using Joinpoint software version 4.9.0.0 [10, 11]. Using permutation tests to determine the most parsimonious model, these regression models determine the optimal number of joinpoints corresponding to changes in the slope of monthly counts over time. Based on starting and ending months of each segment identified by joinpoints, the monthly percent change (MPC) and 95% confidence interval in well visit count was calculated. Main analyses were conducted based on HEDIS-defined age categories (< 1, 1–4, 5–11, 12–17, 18–39, 40–64, and ≥ 65 years) that differ in guideline concordant, well visit frequency. Supplementary analyses (see Supplementary material) were conducted by gender and age as well as race (White vs. Black) and age. The focus of this analysis is describing temporal changes within age groups; no between group comparisons were made.

## Results

Sample characteristics are shown in Table 1. The majority of patients with a well visit from 7/1/2018 to 6/30/21 were female (56.8%) and White (78.7%). The total number of well visits for the 798,571 patients in this 3-year period was 1,718,917 visits. Mean number of visits per patient was 2.1 (± 1.7) visits. Among all well visits, the most common age category was 40–64 years of age (27.4%).

**Table 1:** Demographic characteristics of patients with ≥ 1 primary healthcare related visit during 7/1/2018 to 6/30/21

*Total Patients*	*n* = *798,571*
Race	
Native Hawaiian/Pacific Islander	836 (0.1%)
American Indian/Alaska Native	3,824 (0.5%)
Asian	14,174 (1.8%)
Black/African American	91,486 (11.5%)
White/Caucasian	628,219 (78.7%)
Multiple races	20,199 (2.5%)
Unknown	39,833 (5.0%)
Sex	
Female	453,829 (56.8%)
Male	344,502 (43.1%)
Unknown	240 (0.03%)
Visits/patient, mean(± sd)	2.1 (± 1.7)
* Total visits*	*n* = *1,718,917*
Age at visit (years)	
< 1	210,873 (12.3%)
1–4	180,085 (10.5%)
5–11	152,856 (8.9%)
12–17	123,065 (7.2%)
18–39	264,426 (15.4%)
40–64	471,080 (27.4%)
≥ 65	316,181 (18.4%)
Unknown	351 (0.02%)

**Fig. 1:**
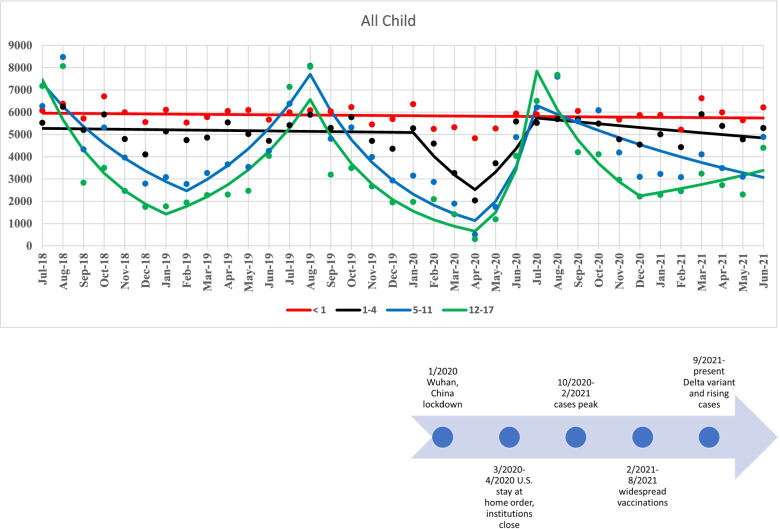
Primary healthcare visits for children < 18 years old. Fitted Joinpoint regression lines

**Fig. 2:**
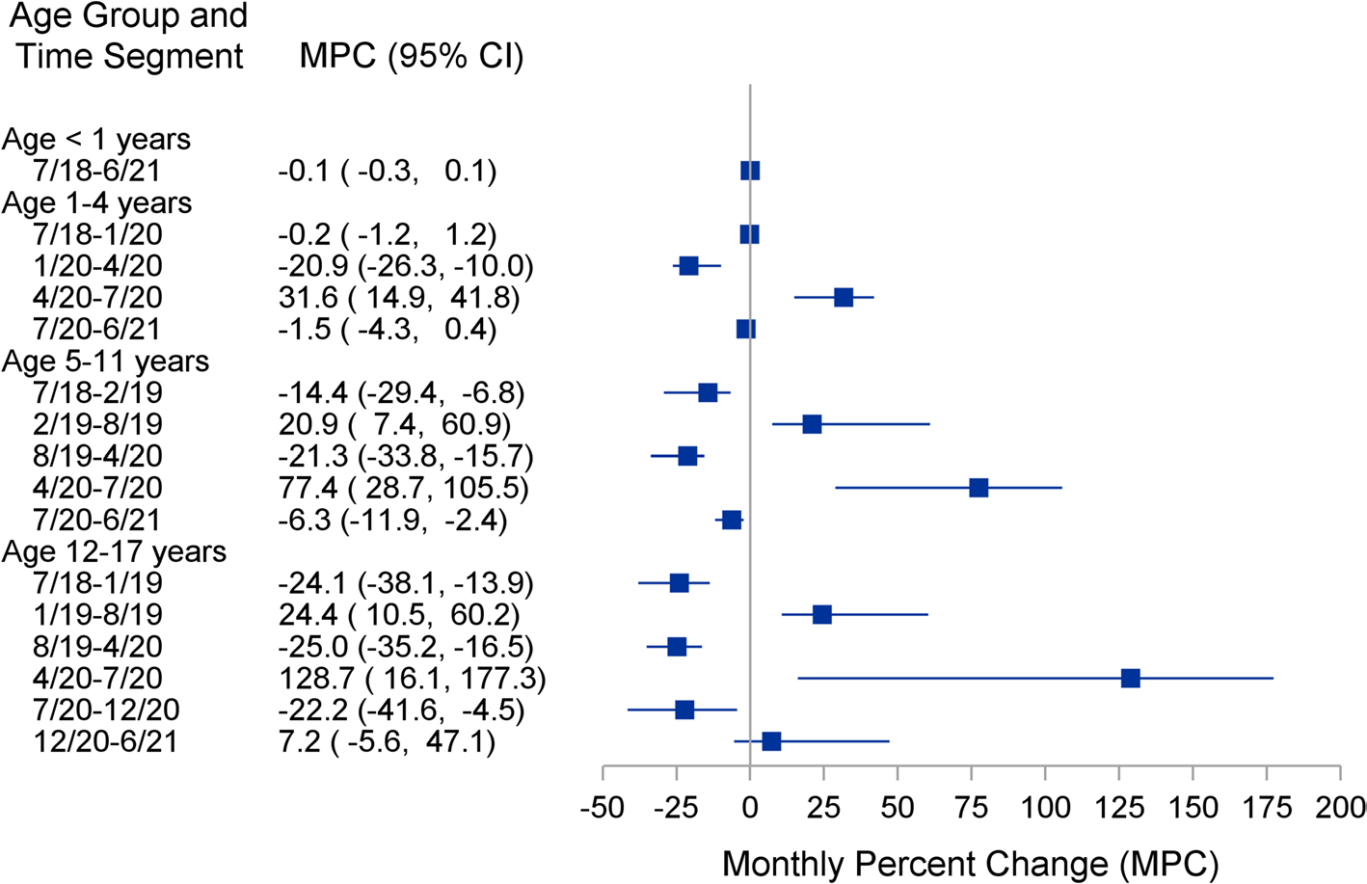
Joinpoint regression estimates - monthly percent change (MPC) and 95% confidence interval for time period segments, children < 18 years old

Well visits per month and fitted joinpoint regression lines from the final model for each child age group are shown in Fig. 1. Corresponding monthly percent change (MPC) with 95% confidence intervals are shown in Fig. 2. For children < 1 year of age, monthly visits remained stable throughout the entire 3-year period (0 joinpoints, MPC = -0.1; 95% CI = -0.3, 0.1). For children 1–4 years old, three joinpoints were identified. There was a significant decrease in well visits from 1/2020 to 4/2020 (MPC = -20.9; 95% CI = -26.3, -10.0) and then a significant increase from 4/2020 to 7/2020 (MPC = 31.6; 95% CI = 14.9, 41.8). Four joinpoints were identified for children ages 5–11; there were alternating periods of significant decreases and increases in monthly visits where the largest decrease in visits occurred from 8/2019 to 4/2020 (MPC = -21.3; 95% CI = -33.8, -15.7) and largest increase occurred from 4/2020 to 7/2020 (MPC = 77.4; 95% CI = 28.7, 105.5). Finally, five joinpoints were identified for patients 12–17 years of age. As with patients 5–11 years of age, there were periods of alternating decreasing and increasing monthly visits. From 8/2019 to 4/2020, visits significantly decreased (MPC = -25.0; 95% CI = -35.2, -16.5). This was followed by an over 100% increase per month in visits from 4/2020 to 7/2020 (MPC = 128.7; 95% CI = 16.1, 177.3).

**Fig. 3:**
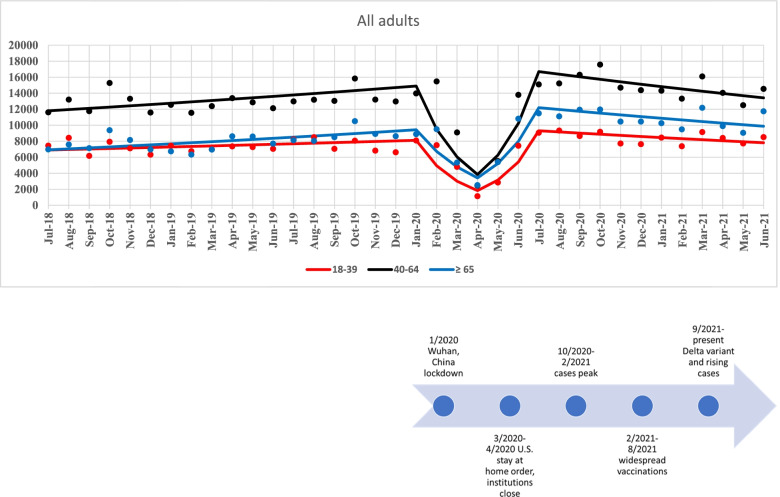
Primary healthcare visits for adults ≥ 18 years old. Fitted Joinpoint regression lines

**Fig. 4:**
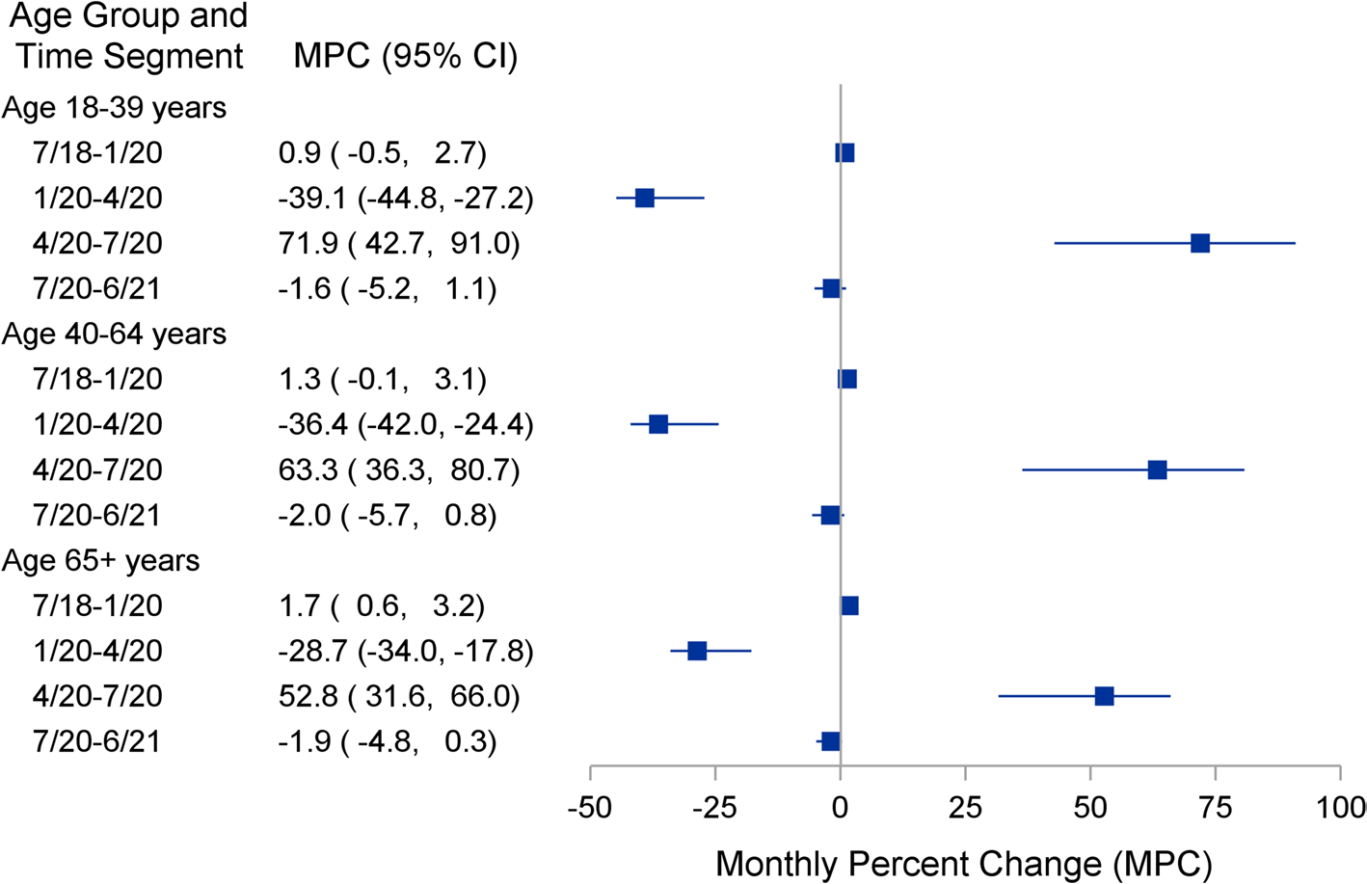
Joinpoint regression estimates - monthly percent change (MPC) and 95% confidence interval for time period segments, adults ≥ 18 years old

Well visits per month, fitted Joinpoint regression lines and MPC estimates for adult age groups are shown in Figs. 3 and 4. For all adult age groups, well visits per month were relatively stable from 7/2018 to 1/2020. In Jan 2020, for each age group, there was a 30–40% decrease in well visits per month until 4/2020. From 4/2020 to 7/2020, there was a 50–70% increase in well visits per month. Finally, from 7/2020 to 6/2021, well visits remained relatively stable for all adult age groups.

As shown in Supplemental Figs. 1, 2, 3, 4, 5, 6, 7 and 8 and e-Tables 2 and 3, change points and patterns of visit decreases and increases among all races and genders were similar for all adult age groups and children ages 0 to < 1 and 1–4 years, relative to overall patient estimates. E-Table 3 and Supplemental Figs. 5, 6, 7 and 8 show similar joinpoints and estimates among female, male, and White children ages 5–11 and 12–17 years relative to overall estimates. Among Black children ages 5–11, joinpoints for the first two segments (prior to 9/2019) were slightly different than among all children aged 5–11 years; but patterns of increasing and decreasing visits were similar. Among Black children aged 12–17 years, four rather than five joinpoints were identified, where compared to all children aged 12–17 years, there was only one segment of decreasing visit counts after 7/2020 (see e-Table 3 and e-Fig. 7).

## Discussion

In a large, multi-state, Midwest health care system, we observed a large, significant decline in well visit utilization after January 2020 to a low point in April 2020 among children 1–4 years of age and among all adults. This decline was followed by a rapid increase after April 2020 and return to well visit utilization rates similar to pre-COVID levels by July 2020. After July 2020, these rates remained stable, as they had been prior to January 2020. Infant (< 12 months of age) remained stable throughout the observation time. For patients between 5 and 17 years of age, it is uncertain how much their variation in well visits was due to the onset of COVID; however, as with adults and children 1–4 years of age, well visits seemed to hit their lowest point in April 2020 before starting to increase to approximate pre-COVID levels in July 2020. This age group experienced peaks prior to the start of the school year when school and sports physicals are mandated. However, from mid-2018 through the end of the year, this age group (5–17 years) had a decline in well visits that hit a low point in January 2019 before increasing again until August 2019 (start of school year), where visits started to decrease. In 2020, the lowest period of utilization was in April, instead of January as in 2019. Well visits continued to decline through the first months of the pandemic among patients 5 to 17 years of age.

Our results indicating no decline for infant (< 1 year of age) well visits is consistent with previous evidence for a less severe decline in well visits among younger compared to older children [2], and confirm a Canadian study that reported minimal change in well child visits during the pandemic as compared to the prior year [7]. This finding highlights the importance of age when considering the impact of the pandemic on well visit use. Macy and colleagues [3] and the Centers for Medicare and Medicaid [9] reported a decline in pediatric well visits among children 2 years and younger. It is possible that further dividing this age group would reveal no change in utilization among patients < 1 year of age, as shown in our study. Our study is also consistent with literature indicating a rapid return to higher levels of health care utilization following the low point in well visits [3].

For patients other than infants, there is more flexibility in when well visits need to occur. In contrast, the lack of a decline in well visits among infants is probably due to the need to obtain appropriate vaccinations and evaluate for normal development within a short period of time. Older patients may avoid well-visits due to fear of infection, which was strongly associated with reduced use of medical care during the first COVID wave [12]. Similarly, a majority of dental patients reported fear of infection during a dental procedure and 62.4% reported willingness to receive treatment only after obtaining COVID vaccination [13]. As the pandemic continues, greater knowledge about risks and ways to prevent infection and growth of telemedicine will likely help normalization use of well-visits and other preventive health care. Even if COVID becomes endemic, there will be future pandemics. Providers should be prepared to offer telemedicine at the start of a new pandemic, until knowledge, education and prevention allow for a safe return to primary care utilization.

### Strengths

Results from our expansive patient population are generalizable to rural and urban areas across the American Midwest. Comparisons in well visit trends by gender and race indicate no differential impact of the COVID-19 pandemic on women vs. men or by minority status. Having observation time through June 2021 provides some evidence that well visits have not been impacted by the Delta variant.

### Limitations

We lack data on patients’ perceptions of the health risks of visiting a physician. We are unable to conclude how much of the decline in well visits was due to patient avoidance as compared to restricted access or adoption of tele-health. We did not measure transitions to tele-health. Similarly, we do not have data to determine why there was a rapid return to pre-pandemic well visit rates. We do not have information on patients’ responses to health care systems implementing social distancing, masking and physical barriers which could contribute to greater willingness to obtain well visits. Findings may not generalize to other regions of the United States or to other countries.

## Conclusions

Well visits for children < 1 year of age remained stable as the COVID-19 pandemic onset in the United States. Although older children and adults had a period of declining use of well visits, this was limited to a few months at the start of the pandemic and followed by a return to pre-pandemic levels and trends. Although speculative, if patients who used well visits prior to the pandemic, resumed well visits following the short period of declining use, it is unlikely that the association between the pandemic and changes in well visits resulted in serious harm.

## Supplementary Information

**Additional file 1.**

## Data Availability

The datasets generated and/or analysed during the current study are not publicly available. Data in the VDW may not be shared outside of Saint Louis University and SSM Health Care per terms of our Data Use Agreement. Programming code for this study can be shared upon request to the corresponding author.
