# Supplementary material for: Infant, pediatric and adult well visit trends before and during the COVID-19 pandemic: a retrospective cohort study
Source: BMC Health Serv Res. 2022 Mar 11;22:328. doi: 10.1186/s12913-022-07719-7 (PMC8916698; doi:10.1186/s12913-022-07719-7)
Supplement: Supplementary file 1 — Additional file 1. [file 12913_2022_7719_MOESM1_ESM.docx]

**SUPPLEMENTARY MATERIAL**

| e-Table 1. Current Procedural Terminology (CPT) codes for well visits by age group | | | |
| --- | --- | --- | --- |
|  | CPT codes | ICD-9 codes | ICD-10 codes |
| Infant  Age 0 to < 1 | 99381  99391 | V20.x  V21.1  V70.0  (Age-appropriate) | Z00.1x  Z00.3  Z76.1  Z76.2  (Age-appropriate) |
| Childhood 1 to 4 years | 99382  99392 |  |  |
| Childhood 5 to 11 years | 99383  99393 |  |  |
| Adolescent 12-17 years | 99384  99394 |  |  |
| Adult 18-39 years | 99385  99395 | V70.0 | Z00.0x |
| Adult 40-64 years | 99386  99396 | V70.0 | Z00.0x |
| Adult ≥ 65 | 99387  99397 | V70.0 | Z00.0x |

| e-Table 2. Results from Joinpoint regression for adults by age, sex, and race - monthly percent change (MPC; 95% Confidence Intervals (CI)) for specified change points | | | | | |
| --- | --- | --- | --- | --- | --- |
|  |  | Female | Male | Black race | White race |
| Age group | Period | MPC (95% CI) | MPC (95% CI) | MPC (95% CI) | MPC (95% CI) |
| 18-39 years | 7/2018-1/2020 | 0.8 (-0.7, 2.7) | 1.1 (-0.2, 2.8) | 0.7 (-0.9, 2.7) | 0.9 (-0.5, 2.7) |
|  | 1/2020-4/2020 | **-40.2 (-46.3, -27.7)** | **-37.6 (-43.0, -26.1)** | **-36.8 (-43.3, -23.1)** | **-39.7 (-45.4, -27.7)** |
|  | 4/2020-7/2020 | **78.5 (46.0, 100.8)** | **60.7 (35.0, 77.2)** | **65.9 (34.7, 86.2)** | **73.6 (43.7, 93.3)** |
|  | 7/2020-6/2021 | -2.1 (-5.9, 0.8) | -0.5 (-4.1, 2.1) | -0.8 (-5.1, 2.2) | -1.8 (-5.5, 0.9) |
|  |  |  |  |  |  |
| 40-64 years | 7/2018-1/2020 | 1.1 (-0.3, 3.0) | **1.6 (0.2, 3.3)** | 1.2 (-0.1, 2.8) | **1.3 (-0.1, 3.1)** |
|  | 1/2020-4/2020 | **-38.6 (-44.4, -26.4)** | **-33.4 (-39.0, -21.5)** | **-35.5 (-40.8, -24.1)** | **-36.5 (-42.2, -24.3)** |
|  | 4/2020-7/2020 | **71.6 (41.8, 91.0)** | **52.9 (28.2, 68.2)** | **60.2 (35.3, 75.9)** | **63.8 (36.1, 81.8)** |
|  | 7/2020-6/2021 | -2.2 (-6.1, 0.6) | -1.6 (-5.3, 1.0) | -0.9 (-4.4, 1.6) | -2.2 (-6.0, 0.6) |
|  |  |  |  |  |  |
| ≥65 years | 7/2018-1/2020 | **1.7 (0.5, 3.2)** | **1.8 (0.7, 3.2)** | **2.1 (1.1, 3.5)** | **1.7 (0.6, 3.2)** |
|  | 1/2020-4/2020 | **-29.6 (--35.1,-18.3**) | **-27.4 (-32.6, -17.1)** | **-25.5 (-30.5, -15.4)** | **-29.0 (-34.4, -18.0)** |
|  | 4/2020-7/2020 | **55.3 (32.6, 69.5)** | **49.5 (29.5, 61.9)** | **42.8 (25.2, 53.8)** | **53.7 (31.9, 67.3)** |
|  | 7/2020-6/2021 | -2.1 (-5.1, 0.2) | -1.7 (-4.5, 0.4) | 0.5 (-2.3, 2.5) | -2.1 (-5.1, 0.1) |
| * Significant MPC’s are in bold | | | | | |

| e-Table 3. Results from Joinpoint regression for children by age, sex, and race - monthly percent change (MPC; 95% Confidence Intervals (CI)) for specified change points | | | | | |
| --- | --- | --- | --- | --- | --- |
|  |  | Female | Male | Black race | White race |
| Age group | Period | MPC (95% CI) | MPC (95% CI) | MPC (95% CI) | MPC (95% CI) |
| 0 - < 1 years | 7/2018-6/2021 | -0.1 (-0.4, 0.1) | -0.1 (-0.3, 0.2) | -0.2 (-0.4, 0.1) | **-0.5 (-0.8, -0.3)** |
|  |  |  |  |  |  |
| 1-4 years | 7/2018-1/2020 | -0.3 (-1.2, 1.1) | -0.1 (-1.1, 1.2) | 0.4 (-0.6, 2.0) | -0.4 (-1.4, 1.0) |
|  | 1/2020-4/2020 | **-20.2 (-25.4, -9.5)** | **-21.5 (-27.1, -10.7)** | **-21.1 (-27.0, -9.2)** | **-20.8 (-26.2, -10.0)** |
|  | 4/2020-7/2020 | **30.7 (14.6, 40.5)** | **32.4 (15.4, 43.0)** | **31.8 (14.1, 42.9)** | **31.5 (15.1, 41.6)** |
|  | 7/2020-6/2021 | -1.6 (-4.4, 0.2) | -1.4 (-4.3, 0.5) | -1.2 (-4.3, 0.8) | **-2.0 (-4.7, -0.1)** |
|  |  |  |  |  |  |
| 5-11 years^a^ | 7/2018-2/2019 | **-14.4 (-28.8, -7.0)** | **-14.4 (-30.2, -6.7)** |  | **-15.2 (--29.1, -7.7)** |
|  | 2/2019-8/2019 | **21.0 (7.7, 59.9)** | **20.8 (6.9, 62.4)** |  | **21.3 (7.5, 62.0)** |
|  | 8/2019-4/2020 | **-21.1 (-32.9, -15.6)** | **-21.6 (-34.9, -15.8)** |  | **-21.5 (-34.2, -15.9)** |
|  | 4/2020-7/2020 | **76.5 (29.1, 103.9)** | **78.3 (27.9, 107.3)** |  | **77.7 (28.8, 106.0)** |
|  | 7/2020-6/2021 | **-6.4 (-11.8, -2.6)** | **-6.2 (-12.0, -2.1)** |  | **-6.8 (-12.3, -2.8)** |
|  |  |  |  |  |  |
|  | 7/2018-12/2018 |  |  | **-19.5 (-45.1, -5.6)** |  |
|  | 12/2018-9/2019 |  |  | **11.7 (3.8, 47.5)** |  |
|  | 9/2019-4/2020 |  |  | **-23.0 (-42.4, -14.7)** |  |
|  | 4/2020-7/2020 |  |  | **81.2 (24.7, 114.1)** |  |
|  | 7/2020-6/2021 |  |  | **-5.9 (-12.5, -1.3)** |  |
|  |  |  |  |  |  |
| 12-17 years^a^ | 7/2018-1/2019 | **-22.9 (-37.9, -12.4)** | **-25.4 (-38.0, -15.7)** |  | **-24.8 (-38.6, -14.3)** |
|  | 1/2019-8/2019 | **22.9 (7.4, 58.6)** | **26.1 (12.9, 59.5)** |  | **25.0 (11.4, 62.8)** |
|  | 8/2019-4/2020 | **-24.1 (-34.8, -0.1)** | **-26.0 (-35.5, -19.4)** |  | **-25.3 (-35.9, -18.0)** |
|  | 4/2020-7/2020 | **120.1 (23.9, 166.6)** | **138.3 (56.0, 188.6)** |  | **132.6 (46.2, 184.1)** |
|  | 7/2020-12/2020 | **-20.7 (-40.0, -5.7)** | **-23.8 (-42.7, -11.4)** |  | **-23.6 (-43.7, -10.1)** |
|  | 12/2020-6/2021 | 6.2 (-7.0, 45.6) | 8.2 (-3.3, 45.5) |  | 8.3 (-3.9, 47.4) |
|  |  |  |  |  |  |
|  | 7/2018-1/2019 |  |  | **-22.2 (-41.6, -11.0)** |  |
|  | 1/2019-8/2019 |  |  | **22.3 (8.7, 73.5)** |  |
|  | 8/2019-4/2020 |  |  | **-23.7 (-40.1, -16.9)** |  |
|  | 4/2020-7/2020 |  |  | **98.0 (34.1, 137.2)** |  |
|  | 7/2020-6/2021 |  |  | **-7.3 (-14.3, -2.4)** |  |
| * Significant MPC’s are in bold, ^a^ Change points identified by Joinpoint regression are different for Black race | | | | | |

**e-Figure 1. Fitted Joinpoint regression lines – Female adults**

**e-Figure 2. Fitted Joinpoint regression lines – Male adults**

**e-Figure 3. Fitted Joinpoint regression lines – Black adults**

**e-Figure 4. Fitted Joinpoint regression lines – White adults**

**e-Figure 5. Fitted Joinpoint regression results – Female children**

**e-Figure 6. Fitted Joinpoint regression results – Male children**

**e-Figure 7. Fitted Joinpoint regression results – Black children**

**e-Figure 8. Fitted Joinpoint regression results – White children**
